# Structural, Functional, and Genetic Changes Surrounding Electrodes Implanted in the Brain

**DOI:** 10.1021/acs.accounts.4c00057

**Published:** 2024-04-17

**Authors:** Bhavna Gupta, Akash Saxena, Mason L. Perillo, Lauren C. Wade-Kleyn, Cort H. Thompson, Erin K. Purcell

**Affiliations:** † Department of Biomedical Engineering, Michigan State University, 775 Woodlot Dr., East Lansing, Michigan 48824, United States; ‡ Neuroscience Program, Michigan State University, 775 Woodlot Dr., East Lansing, Michigan 48824, United States; § Institute for Quantitative Health Science and Engineering, Michigan State University, 775 Woodlot Dr., East Lansing, Michigan 48824, United States; ⊥ Department of Electrical and Computer Engineering, Michigan State University, 775 Woodlot Dr., East Lansing, Michigan 48824, United States

## Abstract

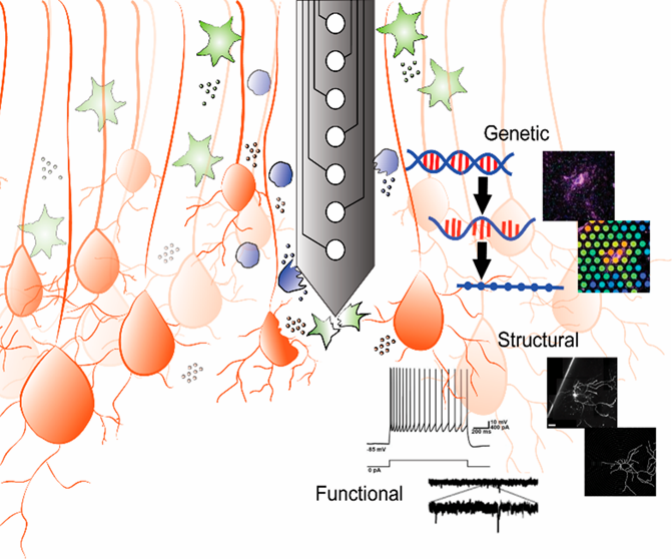

Implantable neurotechnology enables monitoring and stimulating of the brain signals responsible for performing cognitive, motor, and sensory tasks. Electrode arrays implanted in the brain are increasingly used in the clinic to treat a variety of sources of neurological diseases and injuries. However, the implantation of a foreign body typically initiates a tissue response characterized by physical disruption of vasculature and the neuropil as well as the initiation of inflammation and the induction of reactive glial states. Likewise, electrical stimulation can induce damage to the surrounding tissue depending on the intensity and waveform parameters of the applied stimulus. These phenomena, in turn, are likely influenced by the surface chemistry and characteristics of the materials employed, but further information is needed to effectively link the biological responses observed to specific aspects of device design. In order to inform improved design of implantable neurotechnology, we are investigating the basic science principles governing device–tissue integration. We are employing multiple techniques to characterize the structural, functional, and genetic changes that occur in the cells surrounding implanted electrodes. First, we have developed a new “device-in-slice” technique to capture chronically implanted electrodes within thick slices of live rat brain tissue for interrogation with single-cell electrophysiology and two-photon imaging techniques. Our data revealed several new observations of tissue remodeling surrounding devices: (a) there was significant disruption of dendritic arbors in neurons near implants, where losses were driven asymmetrically on the implant-facing side. (b) There was a significant loss of dendritic spine densities in neurons near implants, with a shift toward more immature (nonfunctional) morphologies. (c) There was a reduction in excitatory neurotransmission surrounding implants, as evidenced by a reduction in the frequency of excitatory postsynaptic currents (EPSCs). Lastly, (d) there were changes in the electrophysiological underpinnings of neuronal spiking regularity. In parallel, we initiated new studies to explore changes in gene expression surrounding devices through spatial transcriptomics, which we applied to both recording and stimulating arrays. We found that (a) device implantation is associated with the induction of hundreds of genes associated with neuroinflammation, glial reactivity, oligodendrocyte function, and cellular metabolism and (b) electrical stimulation induces gene expression associated with damage or plasticity in a manner dependent upon the intensity of the applied stimulus. We are currently developing computational analysis tools to distill biomarkers of device–tissue interactions from large transcriptomics data sets. These results improve the current understanding of the biological response to electrodes implanted in the brain while producing new biomarkers for benchmarking the effects of novel electrode designs on responses. As the next generation of neurotechnology is developed, it will be increasingly important to understand the influence of novel materials, surface chemistries, and implant architectures on device performance as well as the relationship with the induction of specific cellular signaling pathways.

## Key References

- GregoryB. A.; ThompsonC. H.; SalatinoJ. W.; RailingM. J.; ZimmermanA. F.; GuptaB.; WilliamsK.; BeattyJ. A.; CoxC. L.; PurcellE. K.Structural and Functional Changes of Deep Layer Pyramidal Neurons Surrounding Microelectrode Arrays Implanted in Rat Motor Cortex. Acta Biomater.2023, 168, 429–43937499727 10.1016/j.actbio.2023.07.027PMC10441615.^1^ The capture of electrodes within thick slices of brain tissue post-implantation enabled electrophysiological and imaging analyses of the damage to neuronal dendritic arbors, changes in dendritic spine morphology, and alterations in synaptic transmission surrounding implanted electrodes in the motor cortex.
- WhitsittQ. A.; KooB.; CelikM. E.; EvansB. M.; WeilandJ. D.; PurcellE. K.Spatial Transcriptomics as a Novel Approach to Redefine Electrical Stimulation Safety. Front. Neurosci.2022, 16, 93792335928007 10.3389/fnins.2022.937923PMC9344921.^2^ The application of new techniques in spatial transcriptomics to the investigation of electrical stimulation effects on brain tissue revealed new sets of genes associated with inflammation, cell cycle progression, and plasticity.
- ThompsonC. H.; SaxenaA.; HeelanN.; SalatinoJ.; PurcellE. K.Spatiotemporal Patterns of Gene Expression around Implanted Silicon Electrode Arrays. J. Neural Eng.2021, 18, 04500510.1088/1741-2552/abf2e6PMC865057033780909.^3^ Bulk RNA sequencing of tissue samples excised surrounding chronically implanted electrodes in motor cortex revealed new sets of differentially expressed genes surrounding electrodes.

## Introduction

Communication in the central nervous system occurs via the widely recognized modalities of electrical and chemical transmission between neurons, and these dynamic interactions generate signals providing information on brain function and behavior.^4^ In recent years, implantable electrodes have enabled the detection and stimulation of neural signals to study and treat a variety of neurological diseases including Alzheimer’s disease, Parkinson’s disease, Huntington’s disease, Tourette’s syndrome, obsessive compulsive disorder, epilepsy, chronic pain, paralysis, and depression.^5−16^ As clinical use expands and interest in nonclinical use of brain implants enters public consciousness, questions related to the safe and ethical use of implanted electrodes have received added attention.^17,18^ Observations of unexplained suboptimal or variable recording performance over chronic implantation periods, shifting stimulation thresholds, and off-target effects of neuromodulation indicate that the understanding of device–tissue interactions remains incomplete.^19−23^ Further characterization of the biological response to implants, as well as definitively ascribing those processes to the design choices (surface chemistry, topography, mechanical characteristics, and feature sizes) that initiate reactivity, is needed in order to achieve a more seamless interface with predictable long-term performance.^24^

Historically, the tissue response to brain implants has been understood through histological assays, which have revealed observations of insertional trauma, inflammation, neuronal loss, and the formation of an encapsulating glial sheath around the inserted probe.^25−29^ Our research focuses on the biological effects of electrode implantation in the brain, and our recent work has uncovered unexpected effects of the device presence on the structure, function, and gene expression of neighboring brain cells.^1,3^ Using whole-cell electrophysiology and two-photon imaging, we observed that implanted recording electrodes are accompanied by reduced excitatory postsynaptic currents, altered dendritic structure, a reduction of spine density, and changes in the spine morphologies and intrinsic excitability of neurons within the recordable radius of implanted electrodes.^1^ In parallel, our transcriptomics data revealed hundreds of differentially expressed genes (DEGs) around the implant site, indicative of disruptions in synaptic transmission, astrogliosis, oligodendrocyte dysfunction, and microglial activation.^3,30^ Spatial transcriptomics on tissue following electrical stimulation showed induction of the genetic signatures of cell death, plasticity, and activity in a manner dependent on the intensity of the applied stimulus.^2^ Recently, our group has implemented computational approaches^31^ with the goal to identify the genes most strongly predictive of changes in recording performance. As the field moves toward smaller and softer devices and the incorporation of nanoscale topologies and nanomaterials, the identification of biomarkers of performance will enable (a) the ability to benchmark the biocompatibility of emerging materials and design parameters in the context of functional outcomes and (b) the identification of biological mechanisms underlying recording quality and stimulation effects, which will enable the design of targeted modifications to improve performance and therapeutic effects.

## Structural and Functional Remodeling Surrounding Brain Implants

The neurons that generate signals that implanted electrodes record or stimulate are structurally complex,^32^ featuring highly branched processes that receive and transmit information-carrying impulses between cells. Dendritic arborization enables efficient sampling of synaptic inputs, including specialized protrusions (spines) that serve as the main site of excitatory synapses.^33^ While a reduction in local neuronal density is commonly observed surrounding devices, the structural and functional impacts of devices on remaining neurons are less clear. We combined two-photon imaging and whole-cell electrophysiology of neurons surrounding electrodes (near-device neurons were within ∼100 μm, and distant-device neurons fell at ∼500 μm from the device) implanted in the rat motor cortex (M1) for up to 6 weeks.^1^ The electrode arrays used were (a) single-shank silicon devices (3 mm shank length, maximum width of 123 μm, and thickness of 15 μm) and (b) polyimide devices (custom designed to match the length and width of the silicon device but with a thickness of 4.4 μm). Based on published literature, the Young’s modulus of polyimide is on the order of one hundredth of that of silicon.^34^ The “device-in-slice” approach captured devices in thick slices of brain tissue, which could then be interrogated by using brain slice electrophysiology and imaging techniques. The results revealed new observations of disrupted dendritic branching, reduced spine densities, and changes in spine morphologies in neurons surrounding devices as well as changes in excitatory neurotransmission and the underpinnings of firing regularity.

**Figure 1:**
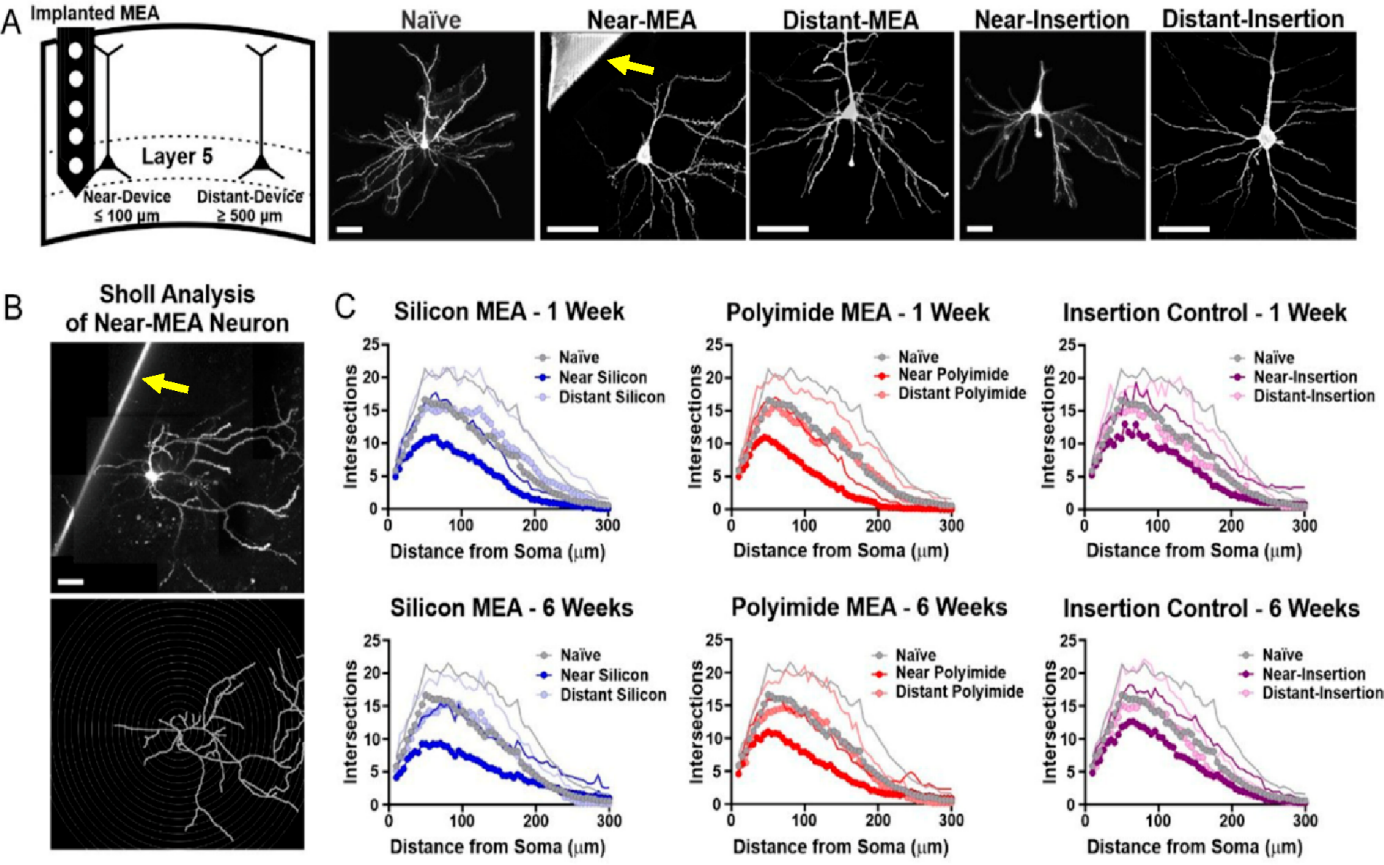
(A, B) Structural changes and (C) reduced dendritic branching in neurons surrounding Michigan-style electrode arrays implanted in rat motor cortex. Neurons near (within ∼100 μm) and far from (at ∼500 μm) the device were studied. Yellow arrows indicate device presence. Near- vs distant-insertion refer to control (sham) condition. Scale bars: 50 μm. Adapted from open-access ref (1). Available under a Creative Commons license CC BY-NC-ND 4.0. Copyright 2023.

**Figure 2:**
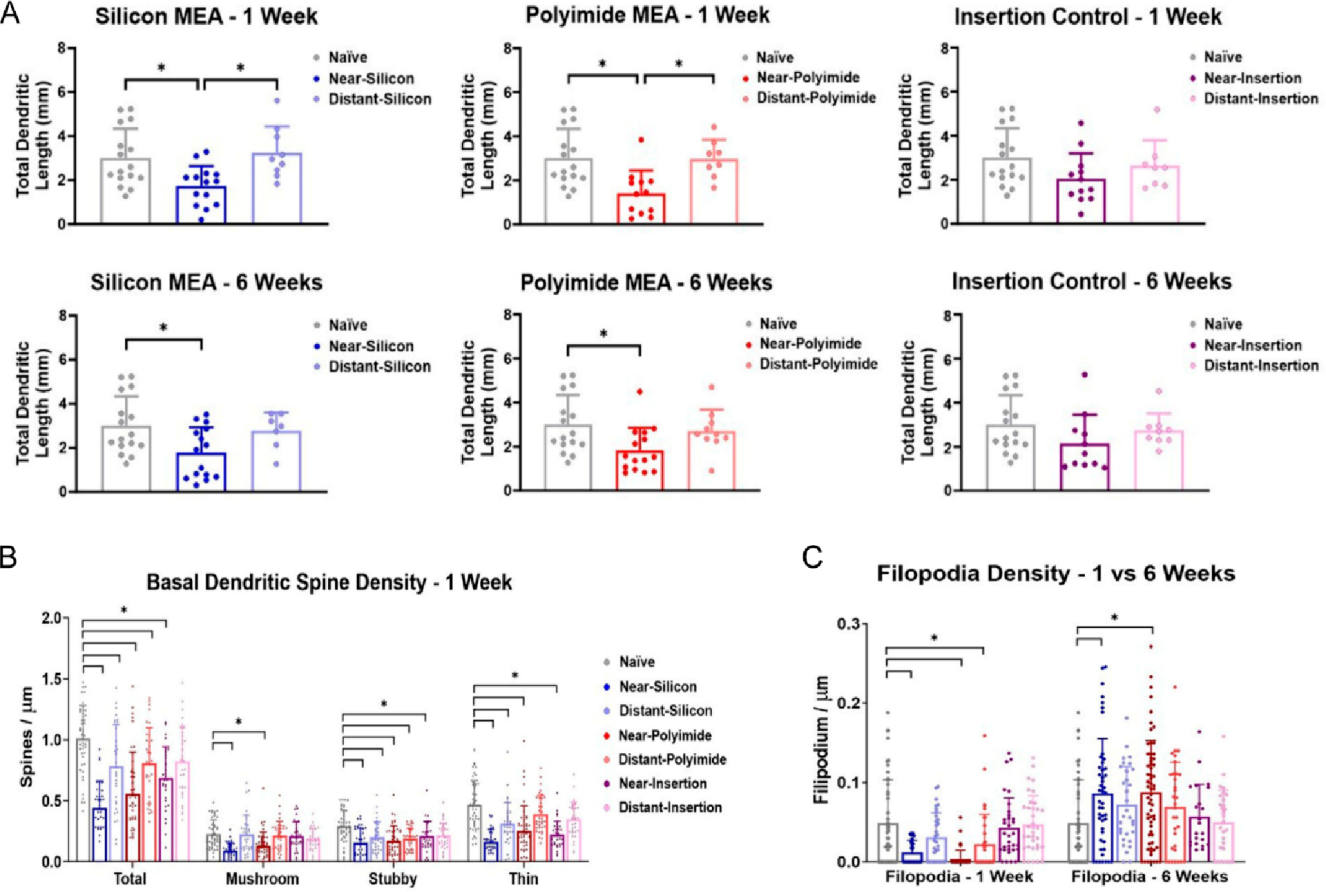
(A) Reduced dendritic length and (B) spine density as well as (C) increased presence of immature spines (filopodia) are evident in neurons near devices over 1 and 6 week time points. Neurons near (within ∼100 μm) and far from (at ∼500 μm) the device were analyzed. Near- vs distant-insertion refer to control (sham) condition. Adapted from open-access ref (1). Available under a Creative Commons license CC BY-NC-ND 4.0. Copyright 2023.

Disruptions to dendritic arborization in neurons near the device were asymmetric, with pronounced losses in dendritic length on the implant-facing side of the neuron (Figure 1A; yellow arrows indicate the device). Sholl analysis, which uses incrementally spaced concentric rings centered at the neuronal soma to map the characteristics of the dendritic arbor,^35^ revealed significantly reduced branching in near-device neurons compared to unimplanted tissue (Figure 1B,C). Dendritic length was significantly reduced at both 1 and 6 week time points in near-device silicon and polyimide devices compared to naïve tissue, and this effect was not visible in sham insertion controls (Figure 2A). Further, spine densities (normalized to dendritic length) were decreased in near-device neurons for both silicon and polyimide devices in comparison with naïve tissue. Additional analysis of spine morphology revealed that mature, functional spine types (mushroom, stubby, and thin morphologies) were significantly decreased at both 1 and 6 week time points in near-device neurons (Figure 2B). On the other hand, the density of filopodia was significantly increased in comparison to naïve tissue (Figure 2C). As filopodia are associated with functional immaturity,^36^ this indicates that neurons surrounding implanted electrodes are relatively disengaged from their native network. Broadly speaking, the reductions in dendritic arborization and spine density around implanted electrodes indicate a decrease in the network input to individual neurons surrounding the electrodes.

**Figure 3:**
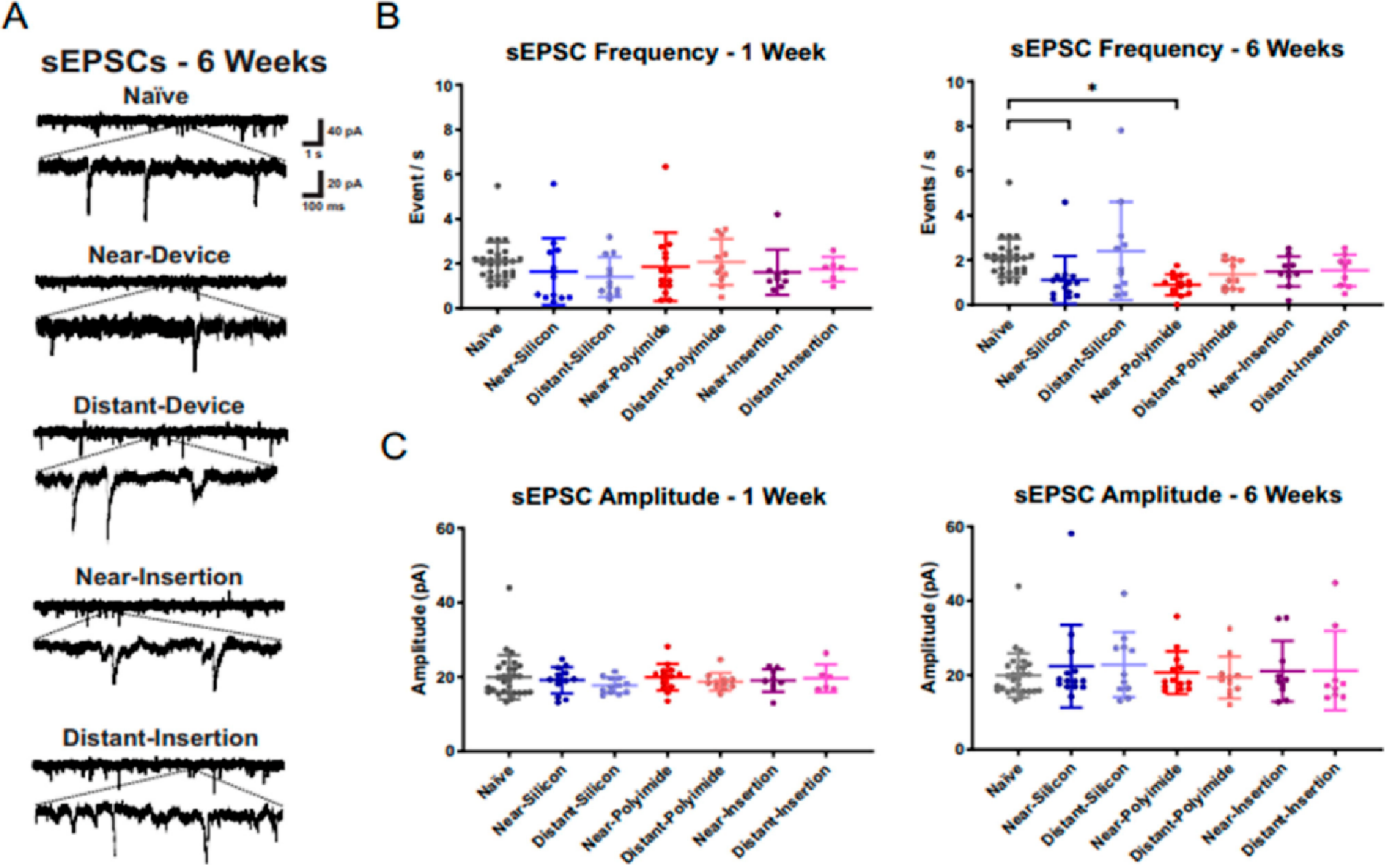
(A) Spontaneous excitatory post synaptic current (sEPSC) frequency is reduced in neurons near devices at 6 weeks (B) without a change in amplitude (C), suggesting functional alterations in the implanted region. Activity of neurons near (within ∼100 μm) and far from (at ∼500 μm) the device were analyzed at both time points. Near- vs distant-insertion refer to control (sham) condition. Adapted from open-access ref (1). Available under a Creative Commons license CC BY-NC-ND 4.0. Copyright 2023.

**Figure 4:**
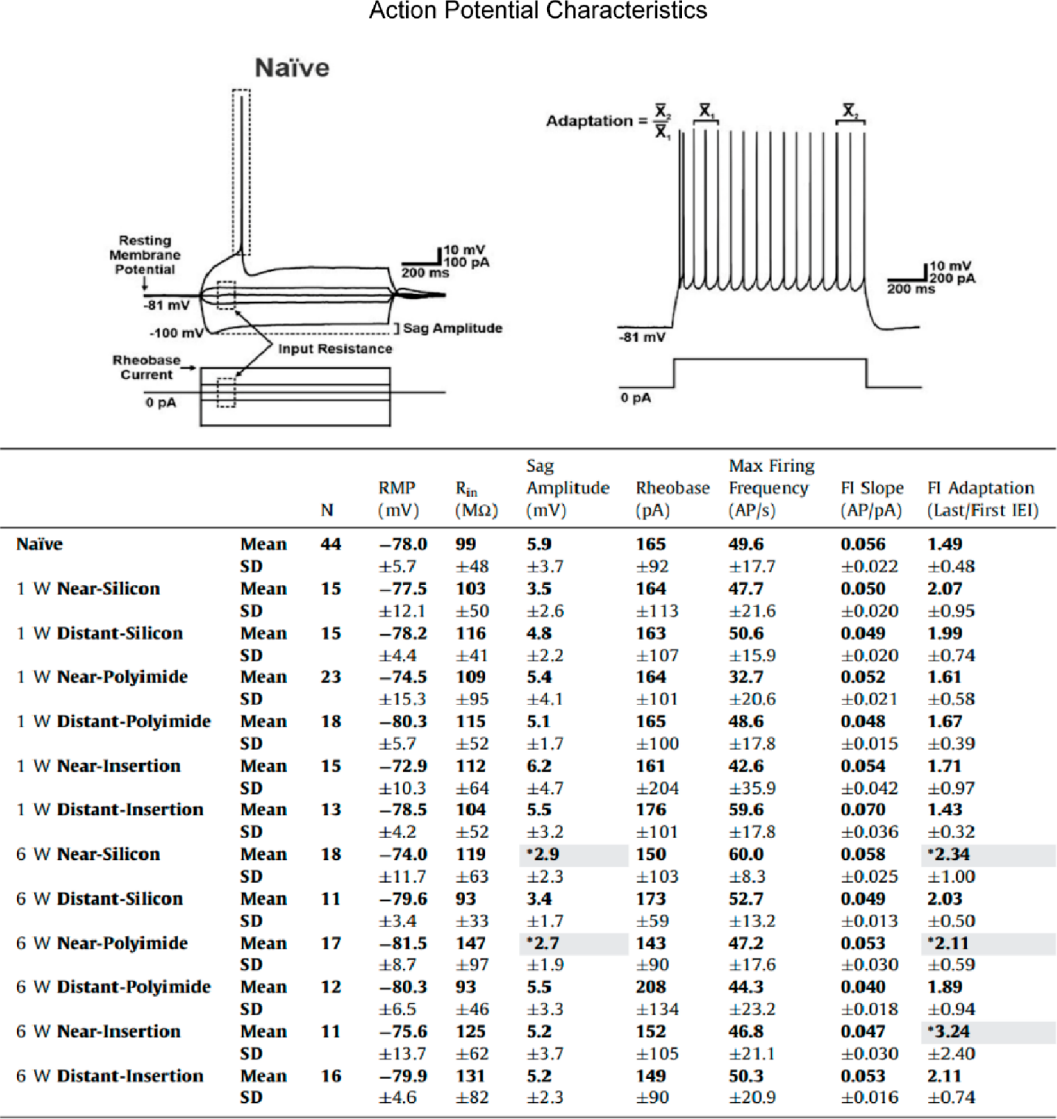
Electrophysiological characteristics of neurons at different time points were analyzed, and a selection is shown. Significantly reduced sag amplitude and increased adaptation are evident in neurons impacted by device presence at 6 weeks vs controls (*P < 0.05, highlighted in gray). Neurons near (within ∼100 μm) and far from (at ∼500 μm) each device type at both time points were analyzed. Near- vs distant-insertion refer to control (sham) condition. Adapted from open-access ref (1). Available under a Creative Commons license CC BY-NC-ND 4.0. Copyright 2023.

Electrophysiological recordings revealed three key functional effects on local neurons at the device interface. First, frequencies of spontaneous excitatory post synaptic currents (sEPSCs), indicative of neurotransmitter release in the absence of an external stimulus, were significantly reduced at the 6 week time point in near-device neurons for both silicon and polyimide implants in comparison to naïve neurons (Figure 3A–C). Second, sag amplitude (i.e., a rebound depolarization in response to a hyperpolarizing stimulus; Figure 4) was significantly reduced at the 6 week time point in near-device neurons in comparison to naïve neurons. Similarly to sEPSC frequency, this effect was absent at the 1 week time point. Third, spike frequency adaptation (characterized by increasing interspike intervals during a constant applied stimulus; Figure 4) was increased in near-device neurons compared to in naïve neurons at the 6 week time point. We hypothesize that these changes, in combination with the disruptions in dendrites and spines, contribute to the signal loss and instability that often accompany implanted electrodes.^20,21,37,38^

## Transcriptional Changes Surrounding Implanted Electrodes

Histological assessments of neuronal density and astrocytic expression of glial fibrillary acidic protein (GFAP) have traditionally been used to study the biological response and tissue health after device implantation. However, histology is relatively low throughput and provides limited information on the biological mechanisms affecting signal quality.^24^ We are employing RNA sequencing (RNA-seq) and spatial transcriptomics techniques to map transcriptional changes in cells surrounding devices,^39^ which has revealed new information about both recording and stimulating electrodes.^2,3,30,40^

**Figure 5:**
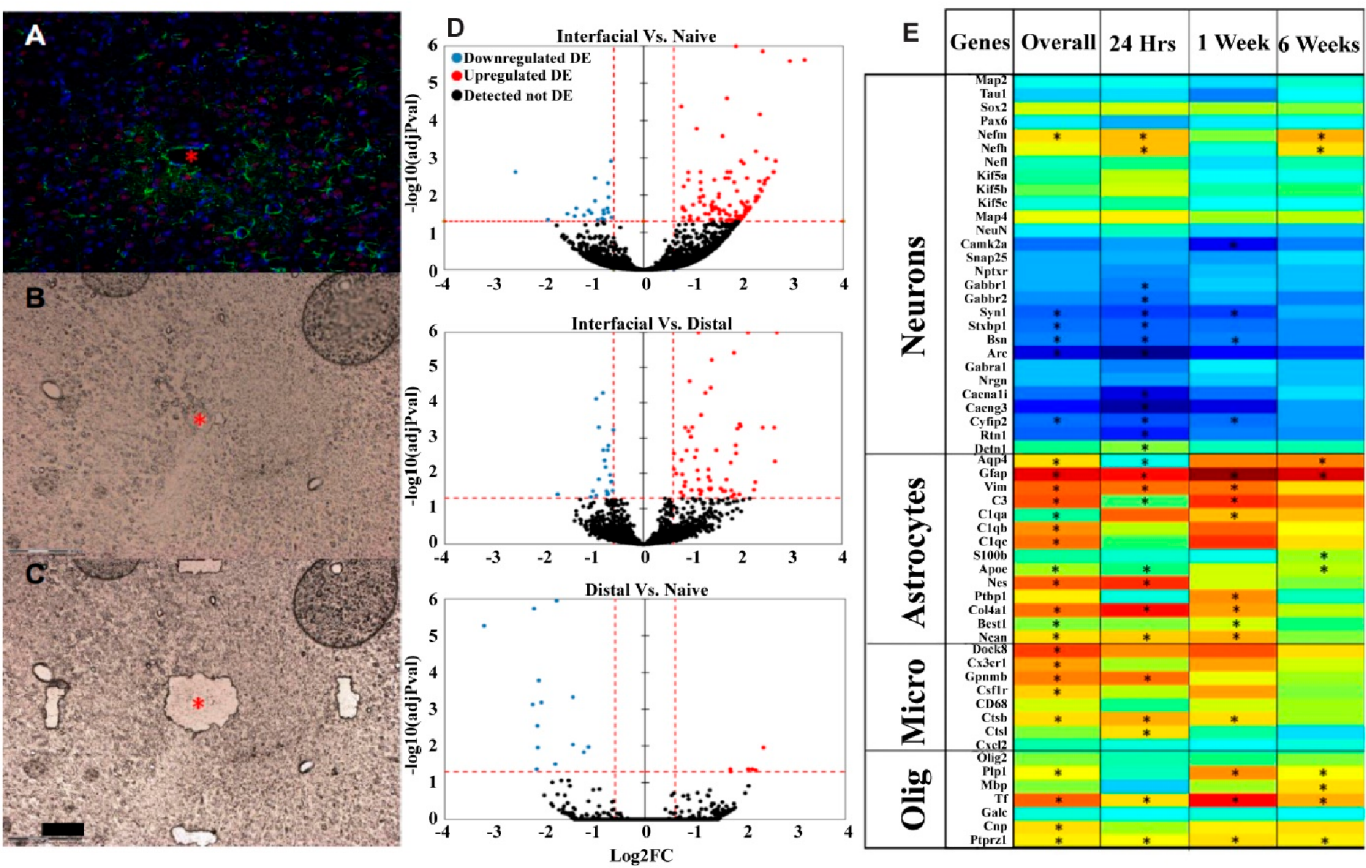
RNA-seq reveals the differential expression (DE) of genes associated with specific cell types surrounding devices. (A–C) Laser capture microscopy was used to collect tissue within 100 μm of or 500 μm away from the device tract (*). Scale bar: 100 μm. (D) Volcano plots illustrate overall DE of genes at near-device relative to naïve tissue (157 DE genes), near relative to far tissue (94 DE genes), and far relative to naïve tissue (21 DE genes). Significance was thresholded at Log2FC ≥ 0.6 and *P* ≤ 0.05 (dashed red lines). (E) Representative heatmap of near vs naïve DE genes for each contrast for individual cell types. Color bar indicates Log2FC. (*) denotes statistically significant DE genes (Log2FC ≥ 0.6 and *P* ≤ 0.05). Adapted from open-access ref (3). Available under a Creative Commons license CC BY-NC-ND 4.0. Copyright 2021.

In our initial study, RNA sequencing of interfacial (≤100 μm from device site) and distal tissue (∼500 μm from device site) samples collected via laser capture microscopy yielded hundreds of DEGs in tissue proximal to implanted electrodes relative to non-implanted naïve tissues (Figure 5).^3^ Genes were grouped based on previously identified associations of cellular interactions and expression (Figure 5E). Mainly, the subsets showed significant DEGs associated with neuronal function and plasticity (e.g., *Camk2a* and *Arc*), astrocyte activation and fibrosis (e.g., *Aqp4*, *Gfap*, *Vim*, *Best1*, and *Ptbp1*), reactive microglia/inflammation (e,g., *Cx3cr1*, *Tnfrsf1a*, *Gpnmb*, and *C3*), phagocytosis (*Dock8*), proliferation (*Csf1r*), lysosomal activity (*Ctsb* and *Ctss*), and oligodendrocyte metabolism and myelin maintenance (e.g., *Plp1*, *Mbp*, *Cnp*, *Tf*, and *Fth1*).

These RNA-seq results add to the current knowledge of gene expression changes involved in the tissue response to implanted electrodes in the brain.^41−44^ For example, reactive microglia continue to highly express inflammatory and phagocytic genes out to 6 weeks post-implantation in our data, possibly reinforcing known neurotoxic roles. A progressive upregulation in oligodendrocyte genes associated with myelination, iron metabolism, and cellular identity potentially suggests a continued need for metabolically taxing remyelination and oligodendrocyte turnover at the device interface. These observations align with recent evidence from Kozai and colleagues supporting the importance of oligodendrocyte genes associated with myelination in device–tissue interactions.^45−47^ Downregulations of neuronal genes associated with synaptic function and dendritic spine maintenance also suggest neuron damage- or dysfunction-related mechanisms involved in the deterioration of signal quality. The implications of these DEGs on the tissue response and device longevity require further study to investigate their potential as therapeutic targets or biomarkers for long-term electrode performance.

**Figure 6:**
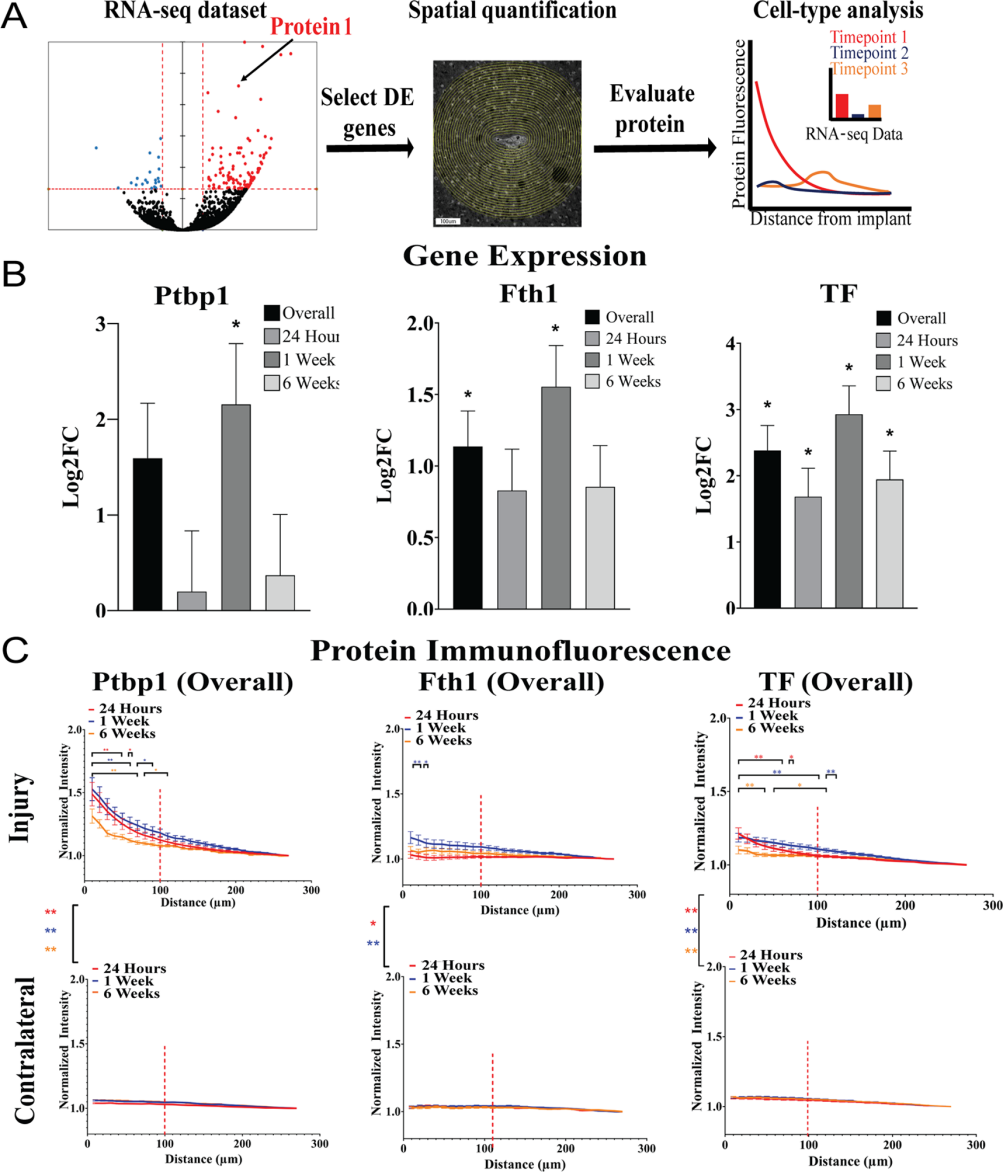
(A) Our workflow identified protein-level changes of markers selected from previous transcriptomics data sets (B) in tissue surrounding implanted electrodes. (C) The quantitative immunohistochemistry results provide information about the spatiotemporal expression of certain proteins in specific cell types. Statistical significance: *P < 0.05 and **P < 0.001. Adapted from open-access ref (40). Available under a Creative Commons license CC BY-NC-ND 4.0. Copyright 2023.

While transcriptomics methods are excellent tools for exploring the biology of the tissue response, mRNA levels alone cannot be used to predict protein expression in the same tissues. Transcriptomics methods can also be prohibitively expensive. In a 2023 study, we sought to identify and quantify protein-level changes using cost-effective, standard immunofluorescence techniques for a subset of genes revealed in our prior transcriptomics data.^3,40^ We evaluated several proteins (GFAP, Nefh, Mbp, Plp1, Ptbp1, Tf, and Fth1) that were found to be differentially expressed at the mRNA level around implanted electrodes and investigated possible differences in expression by cell type (Figure 6). We observed the following: (a) progressive elevation of GFAP fluorescence intensity (consistent with glial scarring around devices), (b) significant changes in Nefh, Mbp, and Plp1 (consistent with neuronal remodeling and remyelination), and (c) elevation of iron-binding proteins Tf and Fth1 at specific time points (indicating potential metabolic changes). Polypyrimidine tract-binding protein-1 (Ptbp1) was strongly elevated within astrocyte and microglia populations 1 week post-implantation, implicating the protein’s potential role in glial reactivity. The spatiotemporal changes in Tf and Fth1 expression within neurons, oligodendrocytes, and microglia around implanted devices may indicate the expanded role of iron metabolism in the tissue response. Overall, the results indicate that exploratory RNA-seq data sets can be used to anticipate and validate fluctuations in the expression of proteins in relevant cell types.

**Figure 7:**
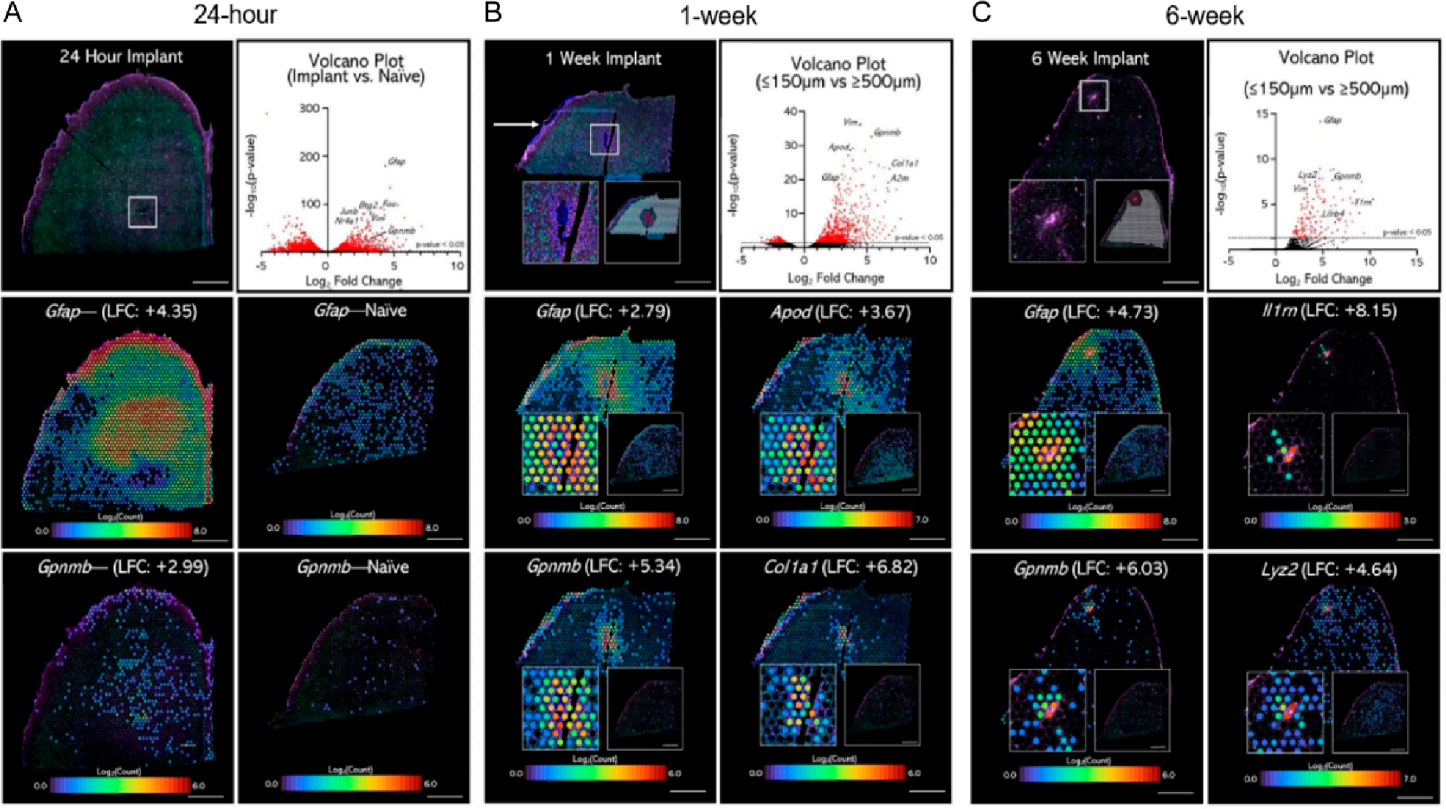
Spatial transcriptomics reveals changes in gene expression in rat primary motor cortex implanted with nonfunctional single-shank silicon Michigan-style microelectrodes for (A) 24 h, (B) 1 week, and (C) 6 weeks. The spatial extent of DEGs is widespread at 24 h and becomes more consolidated over 6 weeks. Scale bars: 1000 μm. Adapted from ref (30). Available under a Creative Commons license CC BY-NC-ND 4.0. Copyright 2021.

Recently, our lab applied a newer spatial transcriptomics method to extend upon our previous findings.^30^ Rats were implanted with nonfunctional single-shank silicon microelectrode arrays in the motor cortex and were sacrificed at 24 h, 1 week, and 6 week time points post-implantation. The spatial transcriptomics assay used (10x Genomics, Visium) enabled fresh frozen tissue to be mounted on microscope slides containing capture sites of spatially barcoded RNA-binding oligonucleotides. Sections were immunostained for neuronal nuclei (NeuN) and GFAP and imaged prior to tissue permeabilization, cDNA synthesis, RNA sequencing, and analysis. We reported (a) the transcriptional profile of single genes with near cellular-scale resolution across entire tissue sections, (b) improved RNA quality by employing fresh frozen tissue samples, and (c) a combination of spatial transcriptomics and quantitative immunohistochemistry on the same tissue slice (Figure 7).

Each time point presented thousands of significant DEGs in comparisons of either implanted vs unimplanted tissue sections (24 h time point; Figure 7A) and areas near (≤150 μm) vs far from (≥500 μm) the device tract (1 and 6 week time points; panels B and C of Figure 7, respectively). A unique observation was that previously reported DEGs extended over a large area of the tissue landscape, over 3.0 mm from the injury site, at 24 h. This became consolidated by 6 weeks, indicating a progression from an acute to chronic foreign body tissue response. Interestingly, the device-reactive astrocytes expressed similar genes to the glia limitans, indicating widespread activation of astrocytes across the cortex. Overall, spatial transcriptomics of the whole device–tissue interface at each time point revealed significant DEGs that could be further analyzed to uncover prominent biological processes at play.

We next sought to extend spatial transcriptomics techniques to profile transcriptional responses in brain tissue receiving intracortical microstimulation (ICMS). Current neuromodulation techniques widely employ electrical stimulation to treat a variety of neurological diseases.^9,12,48−53^ Although previous studies have assessed the biocompatibility of neural stimulation through traditional immunohistochemistry,^54−57^ an explicit understanding of the cellular and molecular mechanisms guiding the effects of electrical stimulation on brain tissue is yet to be completely characterized. Further, the principles guiding the development of safe stimulation protocols may require revision, particularly for newer, small-dimension microelectrodes.^58^ The Shannon equation has been utilized over the last the three decades to associate electrical stimulation intensity with tissue damage thresholds.^59^ New techniques in spatial transcriptomics offer an opportunity to extend upon the Shannon equation, which was founded on traditional histopathological assessments of tissue and accounts for only two parameters of a single pulse (charge and charge density).

We simultaneously conducted spatial transcriptomics (ST) and quantitative immunohistochemistry (IHC) within a tissue section receiving ICMS.^2^ Two types of microelectrode arrays (MEAs) were used: (a) a traditional-style microprobes microwire array (MWA) with five 50 μm diameter platinum–iridium (PtIr) wires (∼2000 μm^2^ geometric area) and (b) a next-generation high-density carbon fiber (HDCF) array with five 6.8 μm diameter carbon fibers containing conically sharpened tips (∼1500 μm^2^ geometric area). Each electrode on both array types was electroplated with PtIr to increase the charge carrying capacity.^60^ Acute and chronic electrode implant procedures were carried out in rat visual cortices (V1).

**Figure 8:**
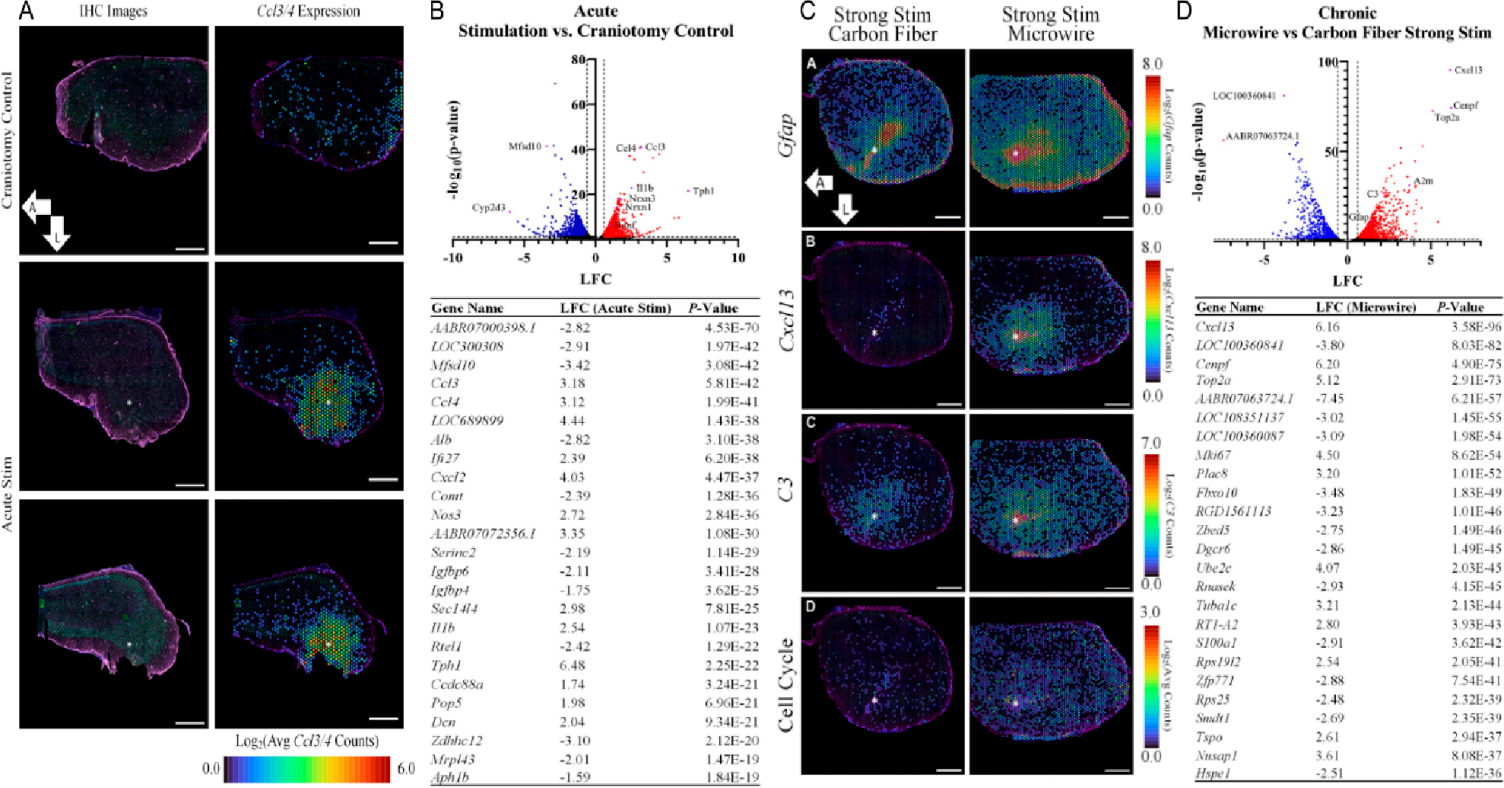
Spatial transcriptomics uncovers DEGs for (A, B) acute and (C, D) chronic stimulating electrode implant conditions. Scale bars: 1000 μm. Adapted from open-access ref (2). Reproduced under a Creative Commons license CC BY-NC-ND 4.0. Copyright 2022.

For acute experiments, an HDCF array was lowered into the V1 region of the visual cortex of an anesthetized male rat and stimulated for 1 h at 25 μA with 200 μs/phase and 50 Hz (charge density = 0.347 mC/cm^2^, charge per phase = 5 nC). A craniotomy control was used for data analysis and comparison. ST uncovered 2914 DEGs for the HDCF stimulated vs control sample (Figure 8A,B). *Ccl3/4* showed pronounced upregulation at the electrode/injury site of the stimulated sample, thereby supporting previously reported increased chemokine expression within hours post-implantation. Overexpression of *Ccl3/4* may be present in extracellular signal-regulated kinase (ERK) signaling for neuronal growth and synapse formation. Interestingly, *Bdnf* and *Nrxn1/3*, genes involved in synaptic formation and plasticity and neurotransmission, were upregulated in the acute experiments. Our previous study conversely found downregulation of *Nrxn3* at the 24 h and 1 week time points with nonfunctional implants,^30^ suggesting that synapse formation or repair could potentially be acute stimulation-specific.

Chronic experiments included one MWA and one HDCF implanted in each V1 hemisphere of five male rats for 4 weeks, and brains were collected 1 day post-stimulation. Seven hour periods of strong (20 nC, 1 mC/cm^2^ for MWA and 14.4 nC, 1 mC/cm^2^ for HDCF) vs weak (2 nC, 0.1 mC/cm^2^ for MWA and 2 nC, 0.13 mC/cm^2^ for HDCF) stimulations were delivered to awake, behaving rats, and the pulses were symmetric, cathodic-first, biphasic, 0.2 ms/phase, and 50 Hz.

Quantitative IHC and transcriptional results were directly compared to reveal a similarity between the GFAP IHC results and spatial expression of *Gfap* in chronic samples. The spatial expression of *Gfap* was more widespread than that revealed by traditional IHC. Prominent expressions of DEGs, namely “cell-killing” (*CxCl13* and *C3*) and cell cycle-related genes, in the MWA condition compared to HDCF were observed (Figure 8C,D). The upregulation of these DEGs indicates that inflammation and cell cycle-associated pathways are more prominently upregulated post-stimulation with MWA compared to HDCF. Generally, strong stimulation induced DEGs involved in cell death and inflammation. Together, these results extend upon previously reported gross histological signs of stimulation-induced tissue damage, with newer observations of more specific underlying cellular and molecular mechanisms. Importantly, the large data sets that were produced lend themselves to further exploration using computational approaches, opening the door to the identification of biomarkers of metrics of interest.

## Connecting Gene Expression with Structural and Functional Changes

We can assimilate our combined observations to develop a more holistic understanding of the influence of devices on the surrounding brain tissue. First, the functional and structural changes observed (Figures 1–4) may be connected. The loss of dendritic length may result in a reduced presence of ion channels that are responsible for hyperpolarization-activated currents, thereby reducing the sag amplitude in these cells. Likewise, since the “pacemaker”-like hyperpolarization-activated currents can promote spiking regularity, increased adaptation in spike trains may be explainable by reduced sag amplitude. Spine density loss and morphological changes could be related to reduced sEPSC frequency since spines are the main excitatory synaptic sites on dendrites. Loss of synaptic contact sites is reinforced by our gene expression data, which revealed reduced expression of the molecular underpinnings of synaptic transmission (e.g., *Camk2a*, *Syn1*, etc.) in near-device tissue^34^ (Figure 5). We have also observed changes in the expression of genes associated with excitability: reduced expression of *Gabbr1* and *Gabbr2* may promote post-insertional hyperexcitability, which may contribute to neuronal cell damage.^34^

The observation that the structural findings emerged by 1 week post-implantation (Figures 1 and 2), while functional effects were not observed until the later 6 week time point (Figures 3 and 4), implies an additional contributor to the functional effects, potentially implicating a role of device-reactive glia in functional remodeling at the electrode site.^29^ This hypothesis is supported by our repeated observation of the upregulation of genes (e.g., *C3* and *Serping1*) linked to reactive astroglial-mediated synaptic loss.^30,34,61,62^ Further, reactive microglia and astrocytes can release cytokines, glutamate, and adenosine triphosphate (ATP) at the injury site, which could promote hyperpolarized postsynaptic contacts via opening of potassium and chlorine channels.^29,63,64^ In summary, the neuronal gene expression results reinforce observations of structural and functional changes in the neurons, while the glial gene expression results point to potential underlying mechanisms.

## Future Directions: Materials-Based Strategies and Identification of Biomarkers of Performance

Ultimately, further understanding of how implantation disrupts or instigates specific biological processes will provide insight into methods to improve device biocompatibility and function. Previous work has shown that device feature size affects the tissue response,^65^ and bending stiffness, which is a product of both device dimensions and Young’s modulus, is a known determinant of the biological response to implanted electrodes.^66^ Reductions in device dimensions have a more profound effect on reducing bending stiffness than reductions in Young’s modulus: miniaturization is a path toward alleviating the device–tissue mismatch in mechanical properties. Emerging approaches, such as the use of nanoscale devices and advanced nanomaterials, may help to reduce the foreign body response. Nanoscale coatings can be used to reduce astrocytic surface coverage and maintain neuronal function near devices: for example, the extremely small topographical features of a nanoporous gold coating can inhibit the focal adhesion formation of adherent cells.^67,68^ Carbon materials are also a popular choice as nanocoatings due to their high conductivity, biocompatibility, and versatility as electrophysiological and/or electrochemical sensors,^69−74^ despite trade-offs in signal instability, etching, and fouling of the carbon material itself.^75−77^ Etching and fouling can pose challenges for chronic electrochemical sensing, as the carbon surface degrades over time with high voltage application,^75,78^ whereas coatings, such as electrodeposited PtIr and poly(3,4-ethylenedioxythiophene) poly(styrenesulfonate) (PEDOT-PSS), can enhance the charge carrying capacity.^60^ Carbon nanotubes (CNTs) have similarly been used as coatings due to their exceptional electrical performance and biocompatibility,^69^ while graphene and nanodiamond have proved useful as biocompatible materials.^79−85^ Nanomaterials also have potential as standalone devices: neuromodulation has been achieved via metallic nanoparticles that can be actuated with magnetic fields, light, or ultrasonic waves. Nonetheless, the safety of these and other emerging materials is a key concern.^86^ One of the challenges in assessing biocompatibility is identifying the most informative benchmarks for assessment: it would be particularly helpful to identify those biomarkers that contain both safety and efficacy information.

Our combined techniques in RNA sequencing, spatial transcriptomics, immunohistochemistry, and electrophysiology each produce highly informative, dense, and large data sets that can be further analyzed to identify biomarkers of device–tissue interaction. The application of computational analysis paves the way for extraction of the most important features in the data set and assessment of the modes of interaction between them. We are applying network analysis to the bulk RNA sequencing results in the Thompson et al.^3^ study to identify relevant gene modules and potential target genes of interest (“hub” genes). Network analysis typically focuses on the identification of genes that are co-expressed, meaning that expression varies across samples in a similar fashion.^87^ Differential co-expression analysis (DCEA) takes this concept a step further by assessing the difference in the co-expression of genes between two different conditions (control and experimental): groups of genes (modules) are considered to be of special interest if their correlation structure strongly differs between the two conditions.^88^

**Figure 9:**
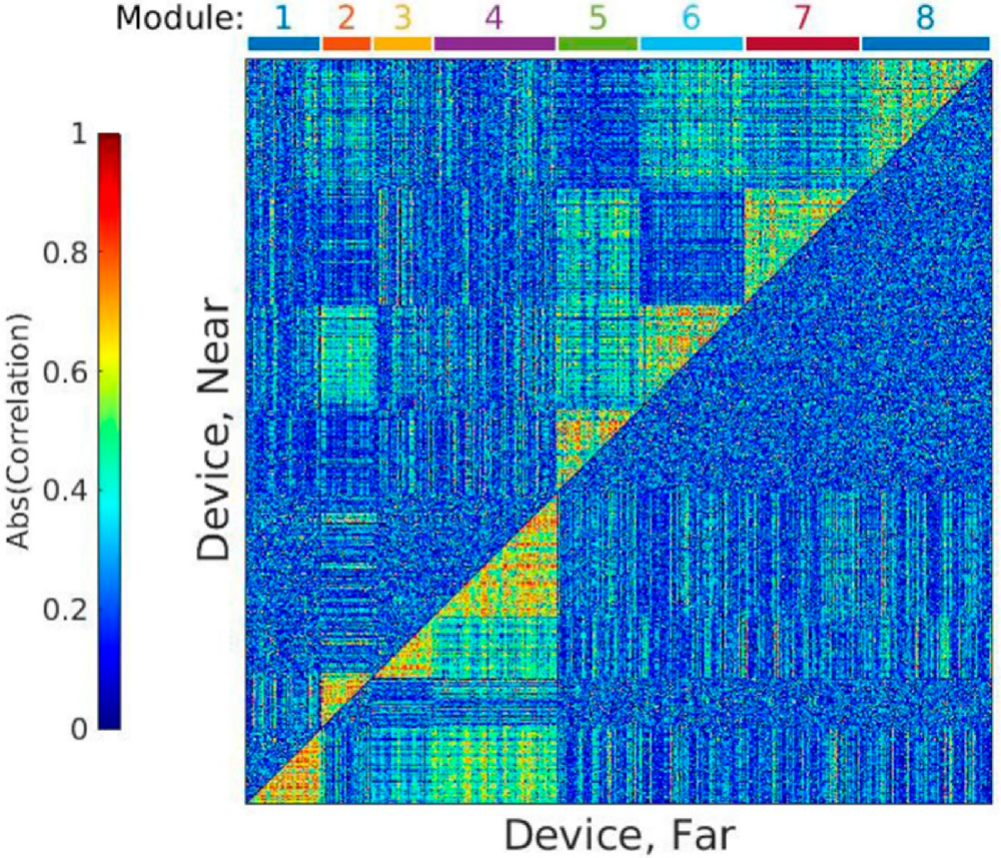
Heatmap displays eight differentially co-expressed gene modules comparing near and far samples in device-implanted rats. The upper diagonal in the heatmap illustrates the correlations between gene pairs in the near-device samples, and the lower diagonal illustrates correlations between gene pairs in the far-device samples. Adapted with permission from ref (31). Copyright 2022. IEEE.

To study the tissue response to neural implants, we used DCEA to reveal modules of genes of interest by applying it on the RNA sequencing data sets near (≤100 μm) and far from (∼500 μm) the implant.^31^ To summarize the pipeline, an adjacency matrix is created, which contains the values of difference of correlation between every gene, near and far from the device. Hierarchical clustering is used to identify modules, which are then sorted in descending order by the median value of the adjacency difference (Figure 9). Key genes of interest (“hub” genes) were obtained by applying principal component analysis (PCA) to the r-log normalized sample counts for the genes in the module. Interestingly, a determinant of dendritic spine morphogenesis (*Kalirin*)^89^ was identified as the top gene of interest in this approach, in alignment with our structural assessments (Figure 2).

Additionally, we are exploring methods to identify biomarkers of signal quality by linking spatial transcriptomics data obtained from the Visium assay with measurements of multi-unit activity (MUA) and the local field potential (LFP). MUA and LFP amplitudes are indicators of single neuron activity and aggregate synaptic activity, respectively.^90^ Briefly, principal component regression (PCR) analysis can be performed for genes and metrics of recording quality (MUA and LFP) and tissue response (GFAP intensity and neuronal density). The log-fold change of DEGs (reflecting the genes that are most strongly affected by the device presence) and the *R*^2^ statistic of the regression (reflecting the strength of the relationship between gene expression and the metric of interest) can be assessed. Using computational tools to link performance metrics with biological pathways of interest is a key current area of inquiry in our research.

## Conclusion

We have taken a multifaceted approach to understand the biological response to implanted electrodes in the brain by employing new techniques to reveal changes in the structure, function, and gene expression of cells surrounding recording and stimulating electrodes. Nonetheless, there are many opportunities to further expand upon our work in future studies. For example, in our structure/function assessments of neurons, we focused our initial characterization on excitatory layer V pyramidal neurons in the motor cortex due to their roles as signal generators for brain–machine interfaces.^91^ However, characterizing the responses of inhibitory neurons is also of great interest for future studies, particularly since alterations in the function of inhibitory neurons could influence the activity of downstream excitatory cells. We are also interested in extending our observation period to longer-term implant durations. Likewise, questions remain regarding the relationship between our data and features of the electrode design. As engineers develop novel materials, surface characteristics, and architectures for implantable neurotechnology as well as new stimulation paradigms, it will be increasingly useful to predict the biological effects of specific modifications. We have produced new data sets with the potential to (a) develop revised computational models of stimulation effects and (b) reveal new biomarkers of device–tissue interaction. Our work is an important step forward in the pursuit of biologically informed device design.
